# Inhibition of epithelial–mesenchymal transition in retinal pigment epithelial cells by a retinoic acid receptor-α agonist

**DOI:** 10.1038/s41598-021-90618-4

**Published:** 2021-06-04

**Authors:** Yuka Kobayashi, Kazuhiro Tokuda, Chiemi Yamashiro, Fumiaki Higashijima, Takuya Yoshimoto, Manami Ota, Tadahiko Ogata, Atsushige Ashimori, Makoto Hatano, Masaaki Kobayashi, Sho-Hei Uchi, Makiko Wakuta, Kazuhiro Kimura

**Affiliations:** Department of Ophthalmology, Yamaguchi University Graduate School of Medicine, 1-1-1 Minami-Kogushi, Ube City, Yamaguchi 755-8505 Japan

**Keywords:** Eye diseases, Medical research

## Abstract

Epithelial–mesenchymal transition (EMT) in retinal pigment epithelial (RPE) cells plays a key role in proliferative retinal diseases such as age-related macular degeneration by contributing to subretinal fibrosis. To investigate the potential role of retinoic acid receptor-α (RAR-α) signaling in this process, we have now examined the effects of the RAR-α agonist Am580 on EMT induced by transforming growth factor-β2 (TGF-β2) in primary mouse RPE cells cultured in a three-dimensional type I collagen gel as well as on subretinal fibrosis in a mouse model. We found that Am580 inhibited TGF-β2-induced collagen gel contraction mediated by RPE cells. It also attenuated the TGF-β2-induced expression of the mesenchymal markers α-smooth muscle actin, fibronectin, and collagen type I; production of pro-matrix metalloproteinase 2 and interleukin-6; expression of the focal adhesion protein paxillin; and phosphorylation of SMAD2 in the cultured RPE cells. Finally, immunofluorescence analysis showed that Am580 suppressed both the TGF-β2-induced translocation of myocardin-related transcription factor-A (MRTF-A) from the cytoplasm to the nucleus of cultured RPE cells as well as subretinal fibrosis triggered by laser-induced photocoagulation in a mouse model. Our observations thus suggest that RAR-α signaling inhibits EMT in RPE cells and might attenuate the development of fibrosis associated with proliferative retinal diseases.

## Introduction

Age-related macular degeneration (AMD) is a serious condition that can lead to blindness. Neovascular AMD (nAMD) is characterized by the invasion of abnormal choroidal neovascularization into the macula and associated fluid leakage^1^. Intravitreal injection of agents that target vascular endothelial growth factor (VEGF) has provided therapeutic benefit in individuals with nAMD by preventing loss of vision or improving visual acuity, although some patients develop subretinal fibrosis and geographic atrophy during such treatment^2,3^. Subretinal fibrosis represents the late stage of nAMD and results in the ablation of retinal structure and vision loss^4^. Various cytokines including interleukin (IL) -6, monocyte chemoattractant protein-1 (MCP-1), and IL-8 as well as growth factors such as transforming growth factor-β (TGF-β) and platelet-derived growth factor (PDGF) are implicated in this process^5^.

Fibrosis is mediated by cells such as fibroblasts, myofibroblasts, and inflammatory cells and is characterized by the excessive deposition of extracellular matrix (ECM) components^6,7^. Myofibroblasts manifest mesenchymal features and play an essential role in the development and progression of fibrosis^8^. Epithelial–mesenchymal transition (EMT) is a cellular process by which epithelial cells develop mesenchymal features including the expression of myofibroblast markers such as α-smooth muscle actin (α-SMA), fibronectin, and vimentin^9^. Myofibroblasts also remodel the ECM in part through expression of matrix metalloproteinases (MMPs) and tissue inhibitors of metalloproteinases (TIMPs) in association with EMT^10^. The major components of ECM in subretinal fibrosis are collagen types I and IV and fibronectin, with smaller amounts of collagen types III, V, and VI also being present^11,12^. In addition, myofibroblasts mediate matrix contraction, with the expression or activation of focal adhesion-related proteins including focal adhesion kinase (FAK), paxillin, and talin contributing to this process^13,14^. TGF-β induces EMT by activating various downstream effectors including mitogen-activated protein kinases (MAPKs), SMAD proteins, phosphoinositide 3-kinase (PI3K), AKT, and myocardin-related transcription factor (MRTF)^15,16^. EMT of retinal pigment epithelial (RPE) cells contributes to the development of fibrosis associated with vitreoretinal diseases such as nAMD, proliferative vitreous retinopathy, and proliferative diabetic retinopathy^17^. Pathological analysis of the fibrous membranes associated with choroidal neovascularization in patients with nAMD has revealed the presence of RPE cells expressing α-SMA^18^.

Retinoic acid (RA) is a metabolic product of vitamin A and possesses various biological activities, with demonstrated roles as an antioxidant, a regulator of cell differentiation and apoptosis, and inhibitor of fibrosis^19,20^. The RA receptor (RAR) family of nuclear receptors includes the isoforms RAR-α, RAR-β, and RAR-γ^21^. RA has been found to suppress TGF-β signaling^22,23^. Indeed, we previously showed that TGF-β-SMAD signaling is suppressed by all-*trans* retinoic acid (ATRA) in Tenon fibroblasts and by an RAR-γ agonist in RPE cells^24,25^. MRTF-A activation also contributes to TGF-β-induced EMT in RPE cells, with an inhibitor of this transcription factor having been found to attenuate subretinal fibrosis in a mouse model^26^.

We have now investigated the role of RAR-α signaling in an in vitro model of fibrotic tissue formation associated with AMD. We thus examined the effect of an RAR-α agonist (Am580) on the TGF-β2-induced contraction of a collagen gel mediated by mouse RPE cells. We also examined the effects of Am580 on expression of the EMT markers α-SMA, fibronectin, and collagen type I; on the production of MMP2 and IL-6; and on translocation of MRTF-A from the cytoplasm to the nucleus induced by TGF-β2 in these cells. In addition, we investigated the effect of Am580 on the development of subretinal fibrosis in vivo with the use of a mouse model of this condition.

## Results

### Effect of an RAR-α agonist on collagen gel contraction mediated by RPE cells

**Figure 1:**
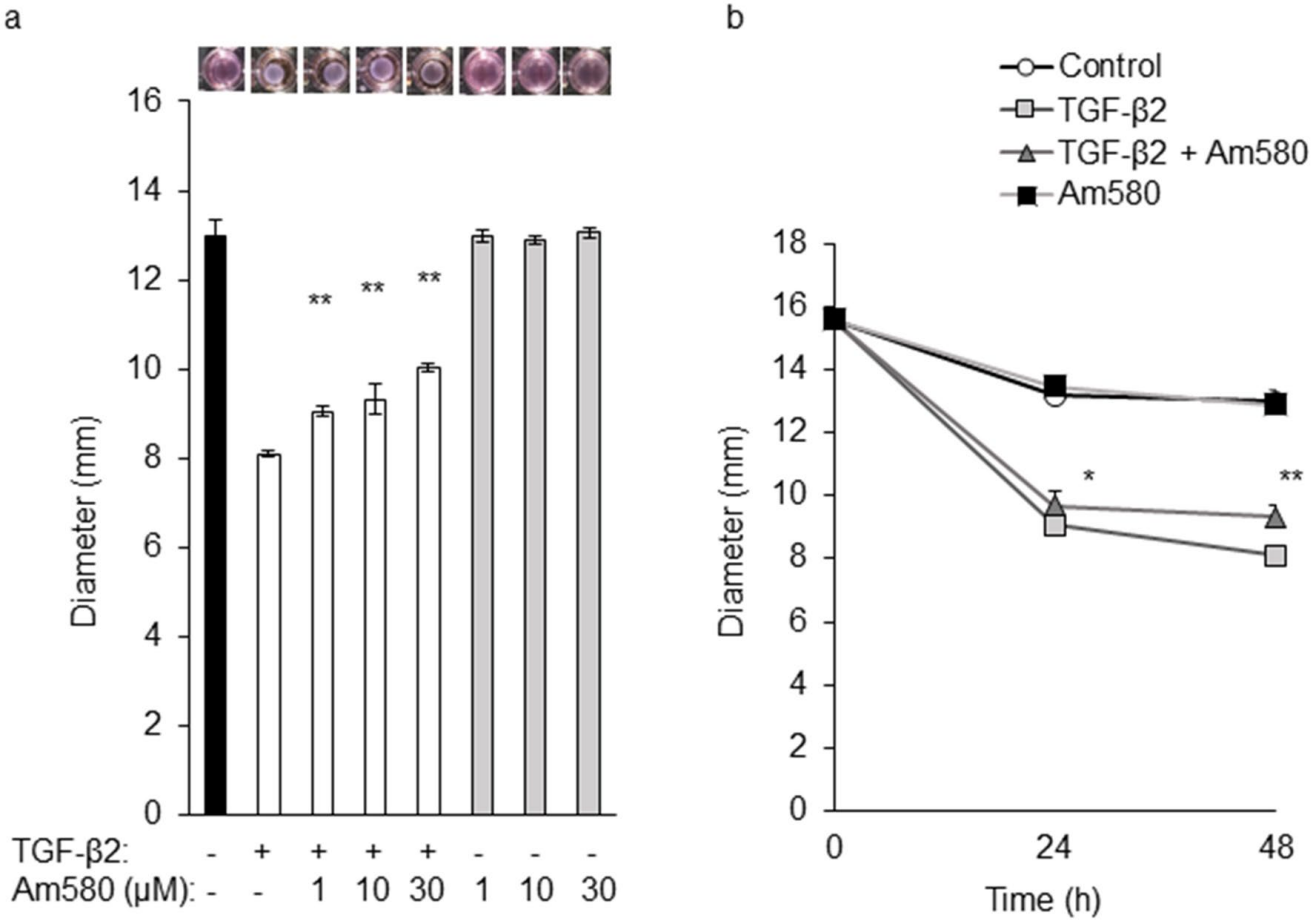
Inhibitory effect of an RAR-α agonist on RPE cell-mediated collagen gel contraction induced by TGF-β2. RPE cells were incubated in collagen gels with or without TGF-β2 (1 ng/ml) and the indicated concentrations (0 to 30 µM) of Am580 for 48 h (**a**) or with or without TGF-β2 (1 ng/ml) and Am580 (10 µM) for 0 to 48 h (**b**), after which the gel diameter was determined. Data are means ± s.d. from four independent experiments. *P < 0.05, **P < 0.01 (Dunnett’s test) versus the corresponding value for cells cultured with TGF-β2 alone.

The collagen gel contraction assay has been studied as an in vitro model of cell-mediated matrix contraction and tissue fibrosis^27,28^. We first examined the effect of the RAR-α agonist Am580 on collagen gel contraction mediated by mouse RPE cells as in vitro model of fibrotic tissue formation associated with AMD. Cells were cultured in collagen gels with or without TGF-β2 (1 ng/ml) and in the presence of various concentrations of the RAR-α agonist for up to 48 h. TGF-β2 induced collagen contraction mediated by the RPE cells, and this effect was inhibited by Am580 in a concentration- and time-dependent manner (Fig. 1). The inhibitory effect of the RAR-α agonist was thus significant at concentrations of ≥ 1 μM and was apparent as early as 24 h. Am580 alone had no effect on cell-mediated collagen gel contraction.

### Effects of Am580 on the expression of EMT markers in RPE cells

**Figure 2:**
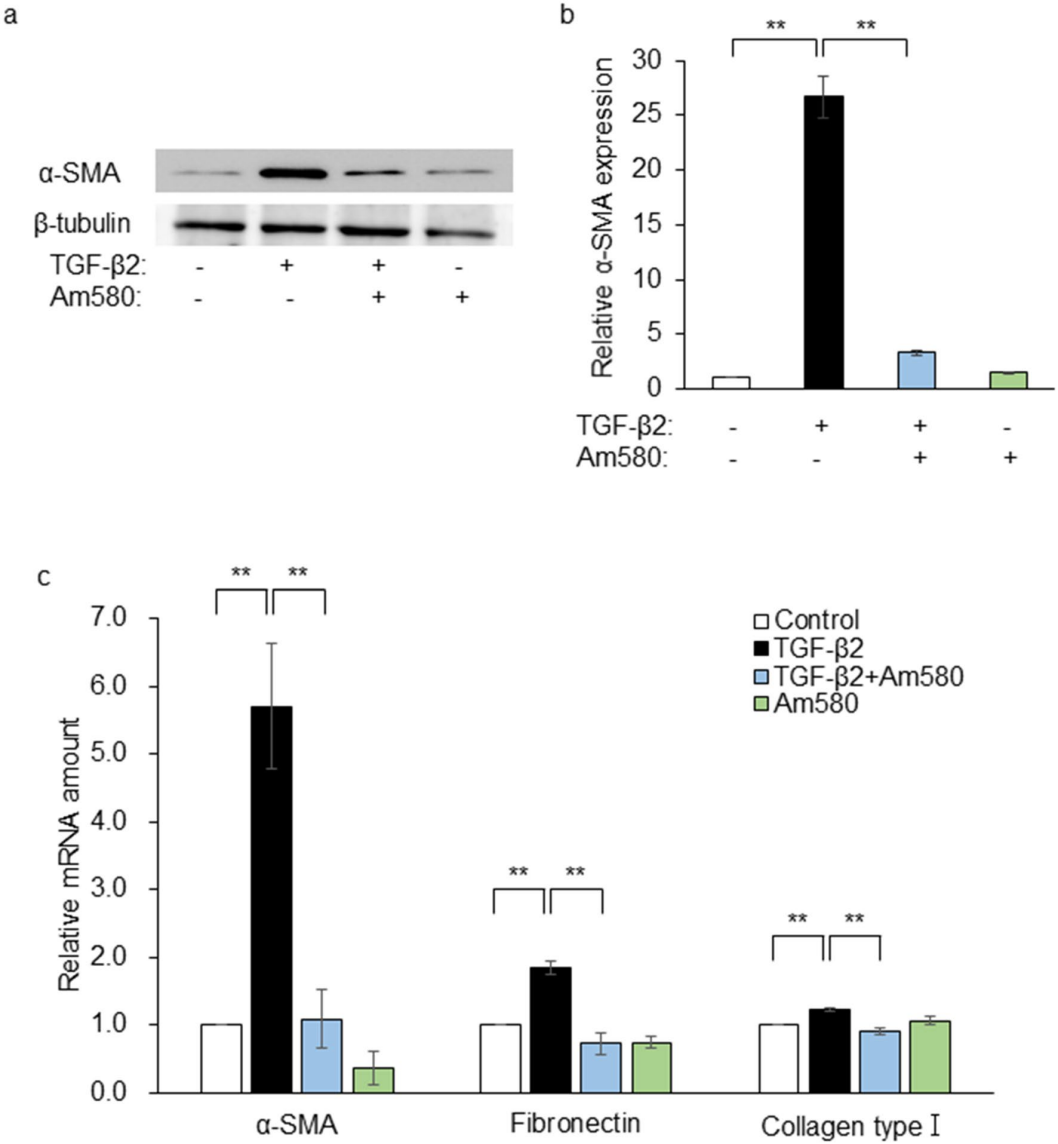
Inhibitory effects of an RAR-α agonist on the TGF-β2-induced expression of EMT markers in RPE cells. (**a**) RPE cells were cultured in collagen gels with or without TGF-β2 (1 ng/ml) and Am580 (10 µM) for 48 h, after which cell lysates were prepared and subjected to immunoblot analysis with antibodies to α-SMA and to β-tubulin (loading control). (**b**) The intensity of each α-SMA band in blots similar to that in (**a**) was normalized by that of the corresponding β-tubulin band, and the normalized values were expressed relative to that for control cells and are presented as means ± s.d from three independent experiments. The intensity of each immunoreactive bands was measured with the use of the Gels commands in ImageJ software. (**c**) Serum-deprived RPE cells were cultured in 24-well plates first with or without Am580 (10 μM) for 6 h and then in the additional absence or presence of TGF-β2 (1 ng/ml) for 48 h, after which the relative abundance of α-SMA, fibronectin, and collagen type I mRNAs was determined by RT-qPCR analysis. Data were normalized by the amount of GAPDH mRNA and are means ± s.d. from three independent experiments. **P < 0.01 (Dunnett’s test).

Subretinal fibrosis is associated with EMT of RPE cells^17^. We therefore examined the effect of the RAR-α agonist on expression of the EMT marker α-SMA in RPE cells cultured in collagen gels. Immunoblot analysis revealed that TGF-β2 increased the abundance of α-SMA in the cells in a manner sensitive to inhibition by Am580 at 10 μM (Fig. 2a,b). Subretinal fibrosis is also accompanied by ECM remodeling. Principal components of the ECM in subretinal fibrosis include collagen type I and fibronectin^12^. We next examined the expression of genes for fibronectin and collagen type I as well as that for the EMT-related protein α-SMA in RPE cells by reverse transcription and quantitative polymerase chain reaction (RT-qPCR) analysis. RPE cells were incubated first with or without Am580 (10 μM) for 6 h and then in the additional absence or presence of TGF-β2 (1 ng/ml) for 48 h. TGF-β2 increased the amounts of α-SMA, fibronectin, and collagen type I mRNAs in the cells, and these changes were inhibited by Am580 (Fig. 2c).

### Effects of Am580 on the expression of pro-MMP2 and TIMP-1 in RPE cells

**Figure 3:**
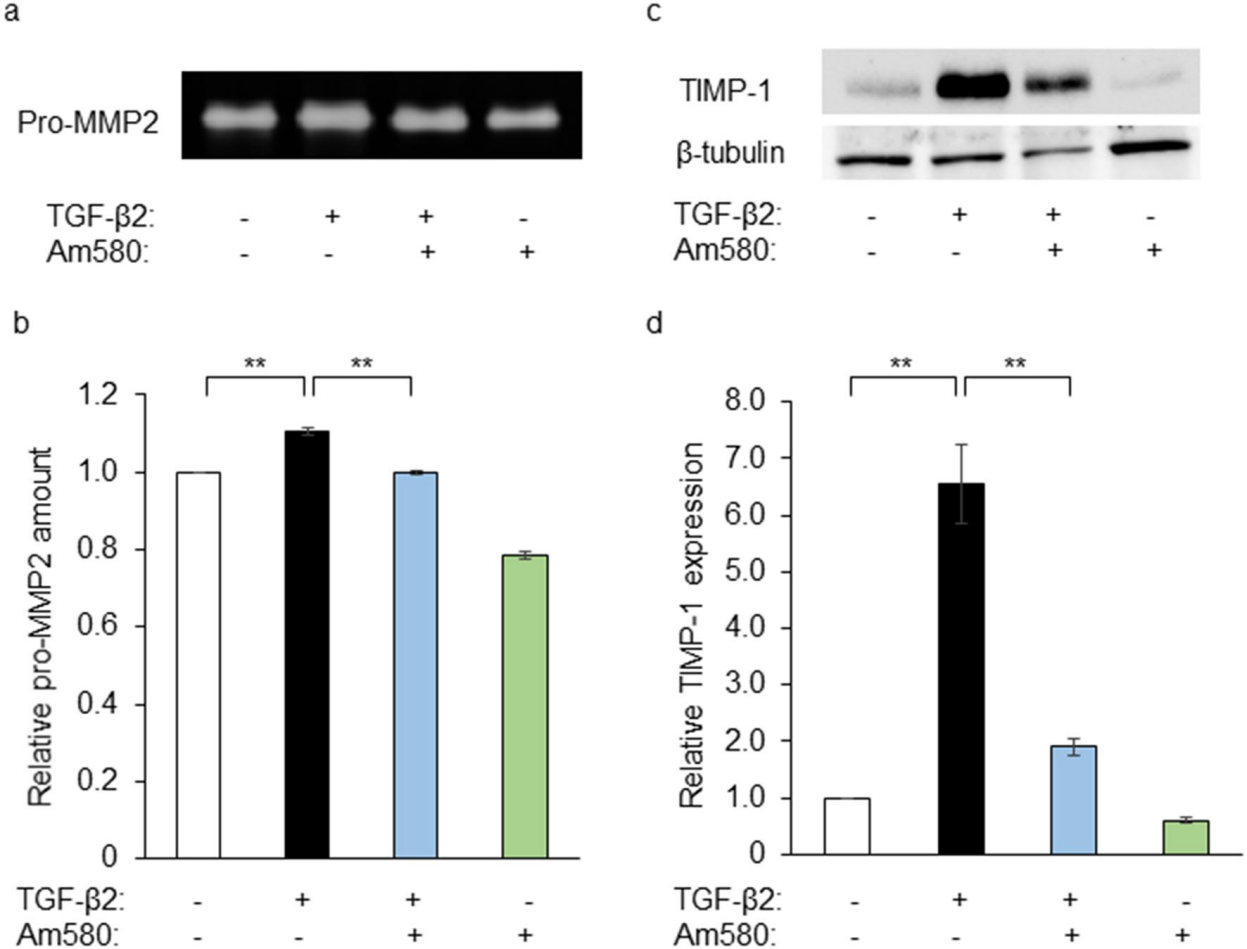
Inhibitory effects of an RAR-α agonist on TGF-β2-induced pro-MMP2 and TIMP-1 expression in RPE cells. (**a**) RPE cells were cultured in collagen gels with or without TGF-β2 (1 ng/ml) and Am580 (10 µM) for 48 h, after which the culture supernatants were subjected to gelatin zymography for detection of pro-MMP2. (**b**) Quantitation of relative pro-MMP2 band intensity for gels similar to that in (**a**). (**c**) RPE cells cultured as in (**a**) were lysed and subjected to immunoblot analysis with antibodies to TIMP-1 and to β-tubulin. (**d**) The intensity of each TIMP-1 band in blots similar to that in (**c**) was normalized by that of the corresponding β-tubulin band, and the normalized values were expressed relative to that for control cells. Quantitative data in (**b**) and (**d**) are means ± s.d. from four independent experiments. The intensity of each bands was measured with the use of the Gels commands in ImageJ software. **P < 0.01 (Dunnett’s test).

Absolute and relative changes in the expression of MMPs and TIMPs play an important role in ECM remodeling and consequent fibrotic tissue formation^10^. We next examined the effects of the RAR-α agonist on the amount of pro-MMP2 released by and the expression of TIMP-1 in RPE cells cultured in collagen gels. Gelatin zymography revealed that TGF-β2 increased the release of pro-MMP2 and that this effect was essentially abolished by Am580 (Fig. 3a,b). Immunoblot analysis also showed that TGF-β2 increased the expression of TIMP-1 in the cells, and again this effect was significantly attenuated by the RAR-α agonist (Fig. 3c,d).

### Effect of Am580 on the expression of paxillin in RPE cells

**Figure 4:**
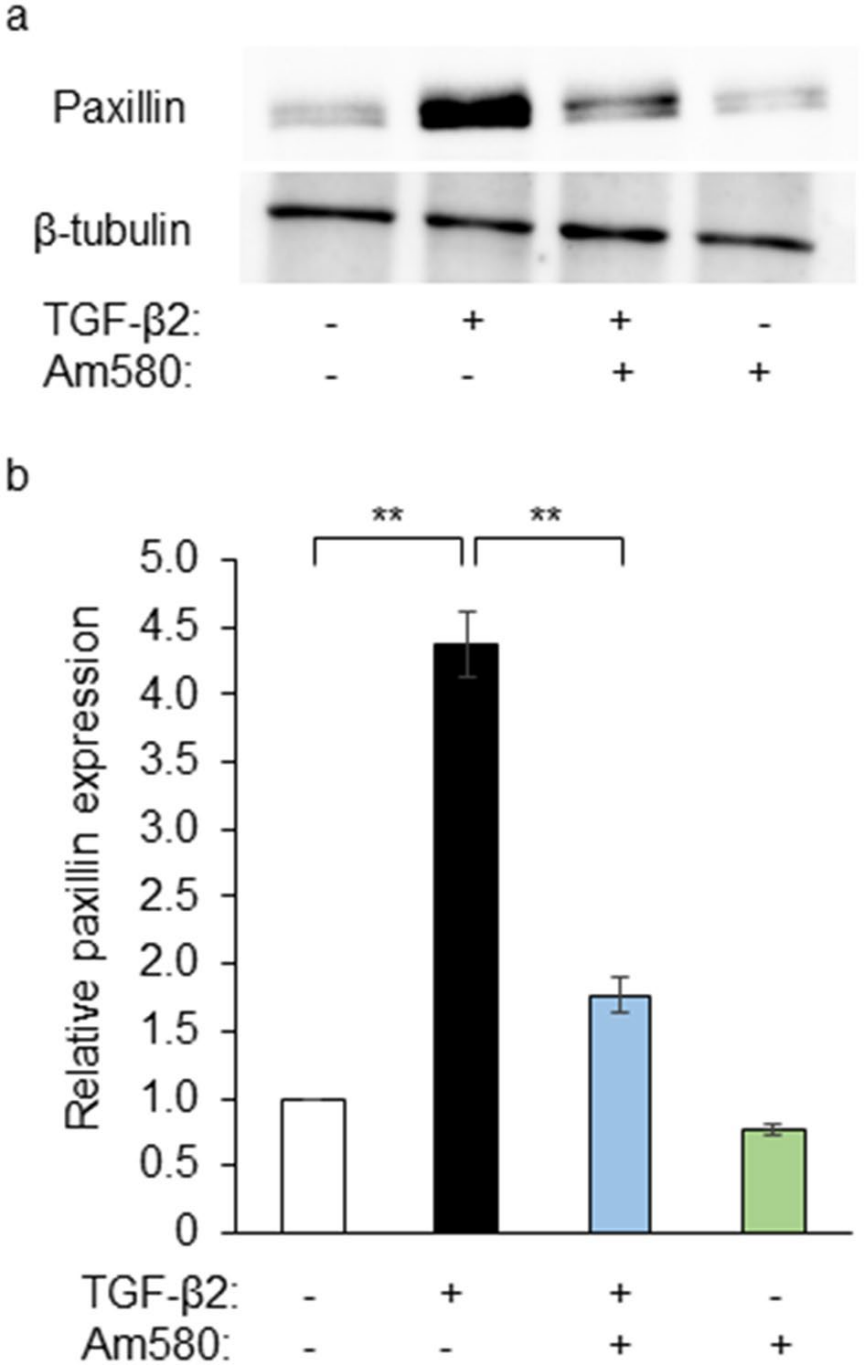
Inhibitory effect of an RAR-α agonist on the TGF-β2-induced expression of paxillin in RPE cells. (**a**) RPE cells were cultured in collagen gels with or without TGF-β2 (1 ng/ml) and Am580 (10 µM) for 48 h, after which cell lysates were prepared and subjected to immunoblot analysis with antibodies to paxillin and to β-tubulin. (**b**) The intensity of each paxillin band in blots similar to that in (**a**) was normalized by that of the corresponding β-tubulin band, and the normalized values were expressed relative to that for control cells and are presented as means ± s.d. for four independent experiments. The intensity of each immunoreactive bands was measured with the use of the Gels commands in ImageJ software. **P < 0.01 (Dunnett’s test).

The expression and activation of focal adhesion-associated proteins contribute to EMT and cell migration^14^. We determined the effect of the RAR-α agonist on expression of the focal adhesion-associated protein paxillin in RPE cells cultured in collagen gels. Immunoblot analysis revealed that TGF-β2 increased paxillin expression and that this effect was markedly inhibited by Am580 at 10 μM (Fig. 4).

### Effect of Am580 on IL-6 release by RPE cells

**Figure 5:**
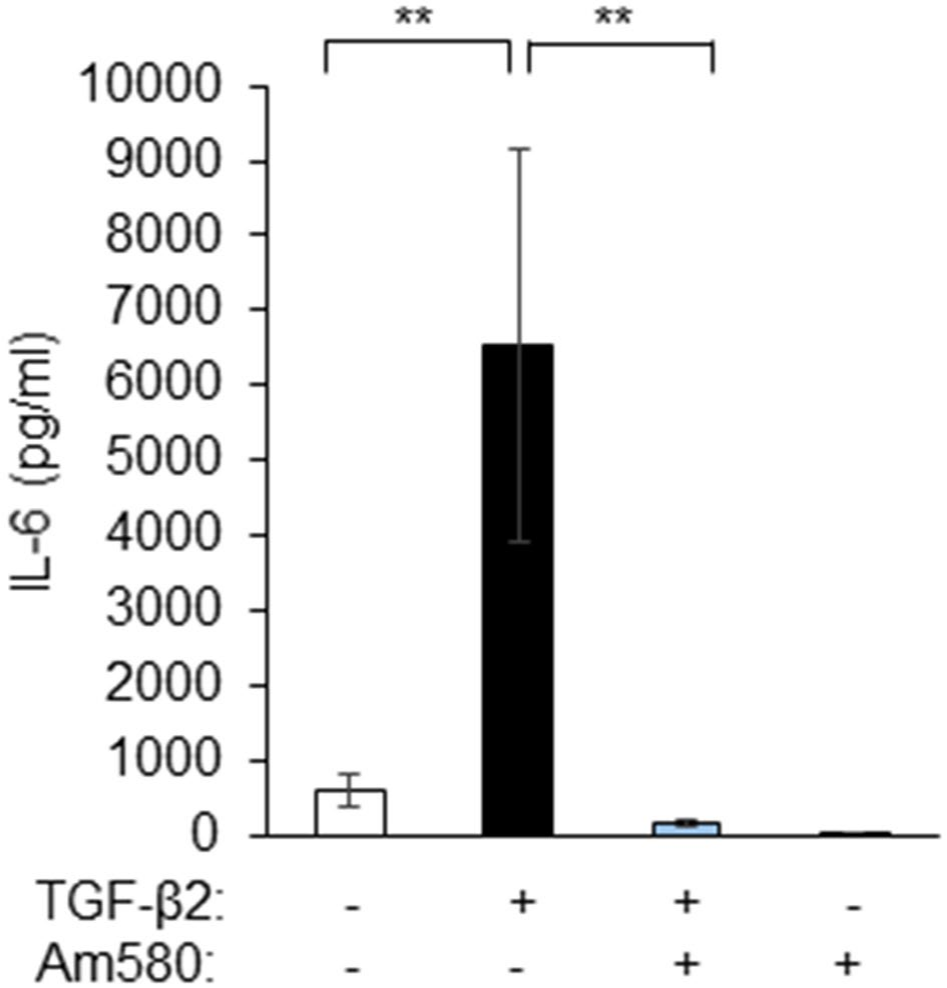
Inhibitory effect of an RAR-α agonist on the TGF-β2-induced release of IL-6 by RPE cells. RPE cells were cultured in collagen gels with or without TGF-β2 (1 ng/ml) and Am580 (10 μM) for 48 h, after which the culture supernatants were assayed for IL-6. Data are means ± s.d. from four independent experiments. **P < 0.01 (Dunnett’s test).

Inflammatory cytokines such as IL-6 are also implicated in the development of subretinal fibrosis^29^. We therefore examined whether the RAR-α agonist might affect production of IL-6 by RPE cells cultured in collagen gels. An enzyme-linked immunosorbent assay (ELISA) showed that TGF-β2 induced the release of IL-6 by these cells, and this effect was prevented by Am580 (Fig. 5).

### Effect of Am580 on SMAD2 phosphorylation in RPE cells

**Figure 6:**
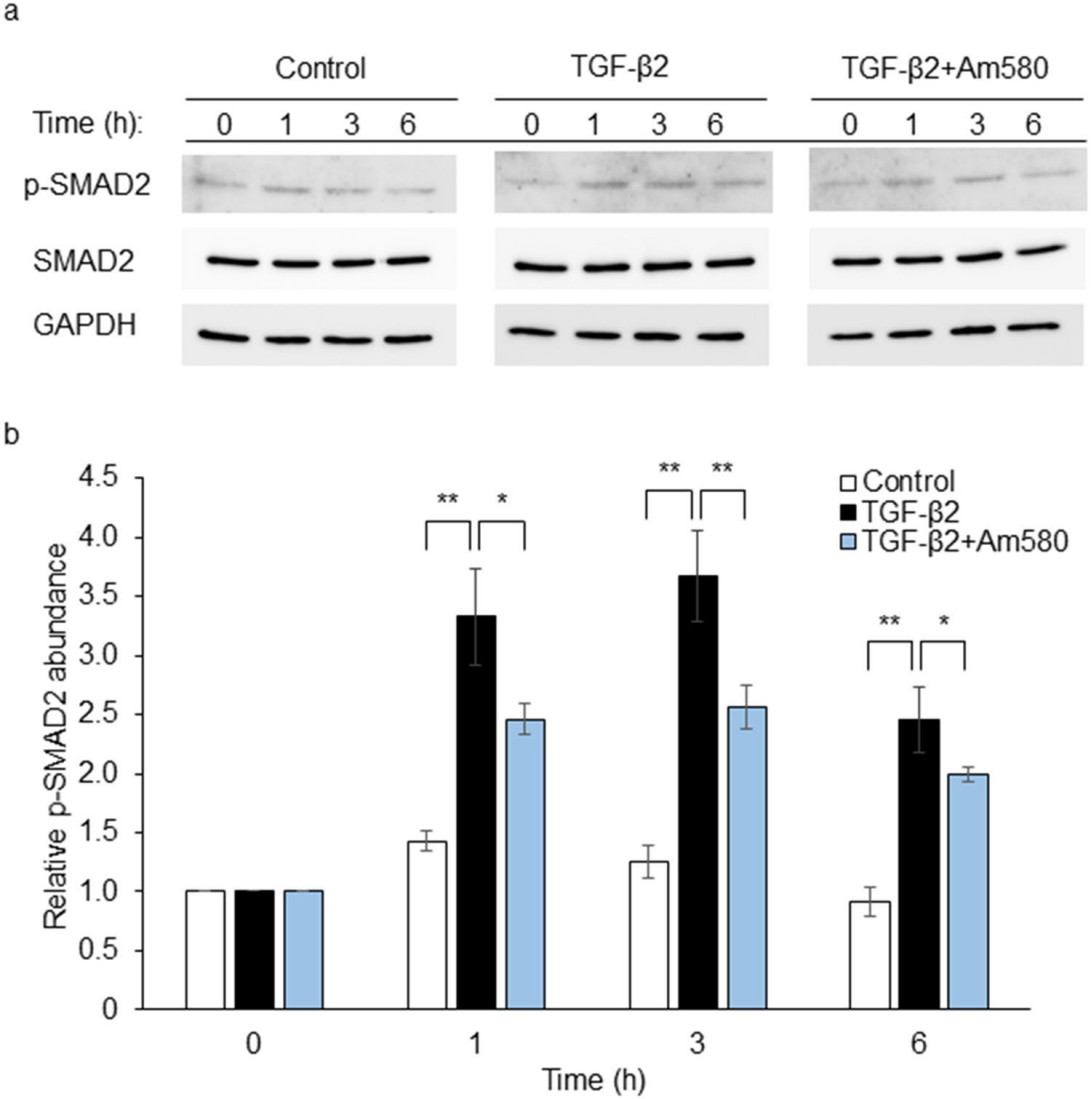
Inhibitory effect of an RAR-α agonist on TGF-β2-induced SMAD2 phosphorylation in RPE cells. (**a**) Serum-deprived RPE cells were cultured in 24-well plates with or without Am580 (10 μM) for 6 h and then in the additional absence or presence of TGF-β2 (1 ng/ml) for the indicated times. Cell lysates were then prepared and subjected to immunoblot analysis with antibodies to total or phosphorylated (p-) forms of SMAD2 and to GAPDH (loading control). (**b**) The intensity of each phospho-SMAD2 band was normalized by that of the corresponding total SMAD2 band, and the normalized values were expressed relative to that for control cells at 0 h. Data are means ± s.d. from four independent experiments. The intensity of each immunoreactive bands was measured with the use of the Gels commands in ImageJ software. *P < 0.05, **P < 0.01 (Dunnett’s test).

TGF-β induces EMT through activation of various downstream signaling pathways including SMAD signaling^15,16^. To examine the effect of the RAR-α agonist on the TGF-β2-SMAD signaling pathway in RPE cells, we determined whether it affects SMAD2 phosphorylation. Immunoblot analysis revealed that TGF-β2 induced the phosphorylation of SMAD2, with this effect being apparent at 1 to 6 h, and that this activation of SMAD2 was suppressed by Am580 (Fig. 6).

### Effect of Am580 on the intracellular distribution of MRTF-A in RPE cells

**Figure 7:**
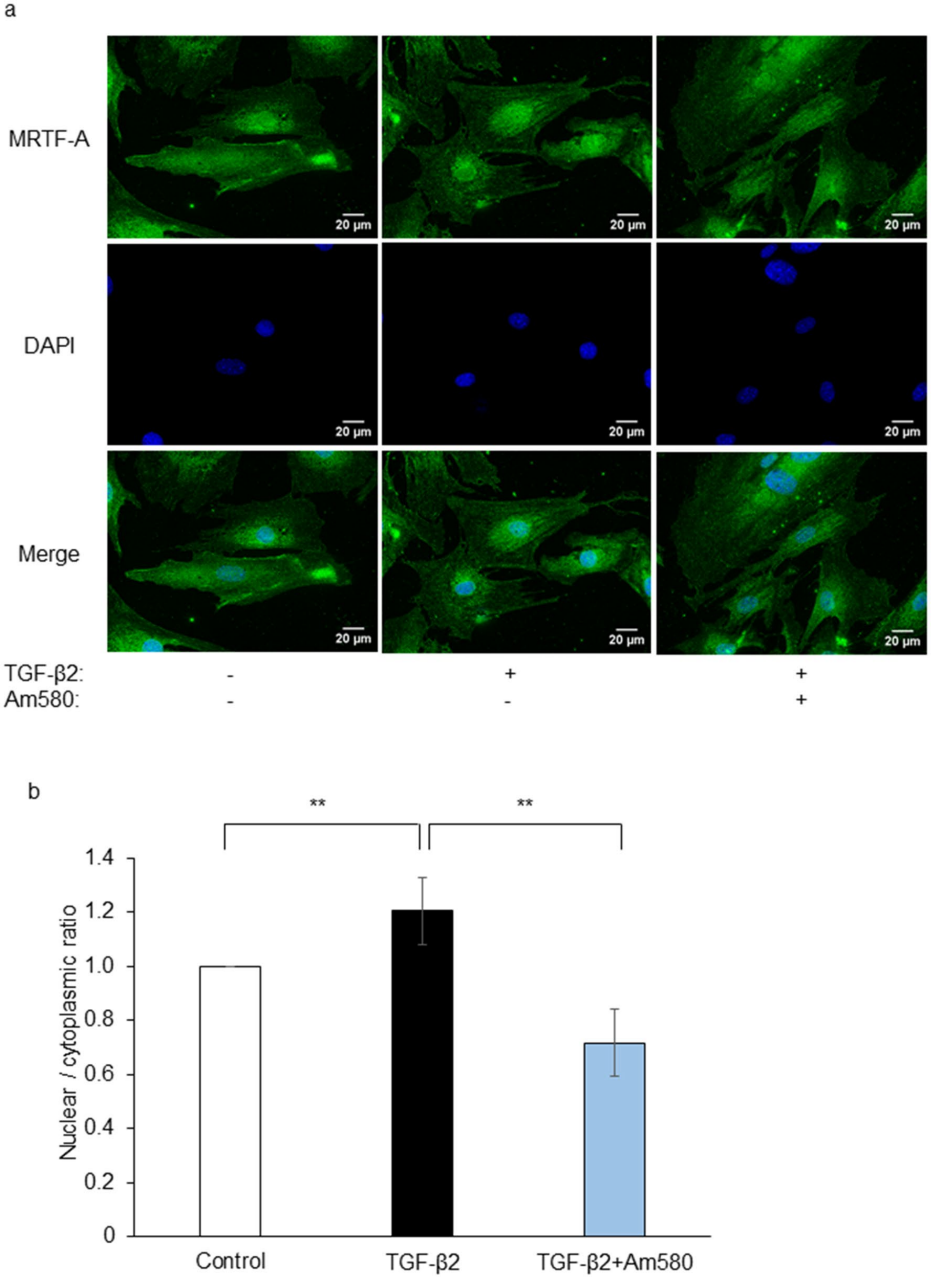
Inhibitory effect of an RAR-α agonist on TGF-β2-induced nuclear translocation of MRTF-A in RPE cells. (**a**) Serum-deprived RPE cells cultured on cover glasses in 24-well plates were incubated with or without Am580 (10 µM) for 12 h and then in the additional absence or presence of TGF-β2 (10 ng/ml) for 24 h, after which they were fixed and subjected to immunofluorescence analysis of MRTF-A (green). Nuclei were stained with DAPI (blue). Scale bars, 20 µm. Data are representative of four independent experiments. (**b**) The nuclear/cytoplasmic ratio of MRTF-A immunofluorescence intensity in experiments as in (**a**) was determined and expressed relative to that for control cells. Data are means ± s.d. for a total of 20 cells in four independent experiments. The intensity of MRTF-A immunofluorescence in the nucleus and cytoplasm was measured with the use of the ROI manager commands in ImageJ software. **P < 0.01 (Dunnett’s test).

MRTF activation contributes to EMT downstream of the TGF-β signaling pathway^25,26^. We also examined the effect of Am580 on the change in the intracellular distribution of MRTF-A induced by TGF-β2 in RPE cells by immunofluorescence analysis. For this experiment, with reference to a previous study, we used TGF-β2 at a concentration of 10 ng/ml in order to induce a readily apparent shift in the distribution of MRTF-A^26^. TGF-β2 induced the translocation of MRTF-A from the cytoplasm to the nucleus, and this effect was attenuated by Am580 (Fig. 7).

### Effect of Am580 in a mouse model of subretinal fibrosis

**Figure 8:**
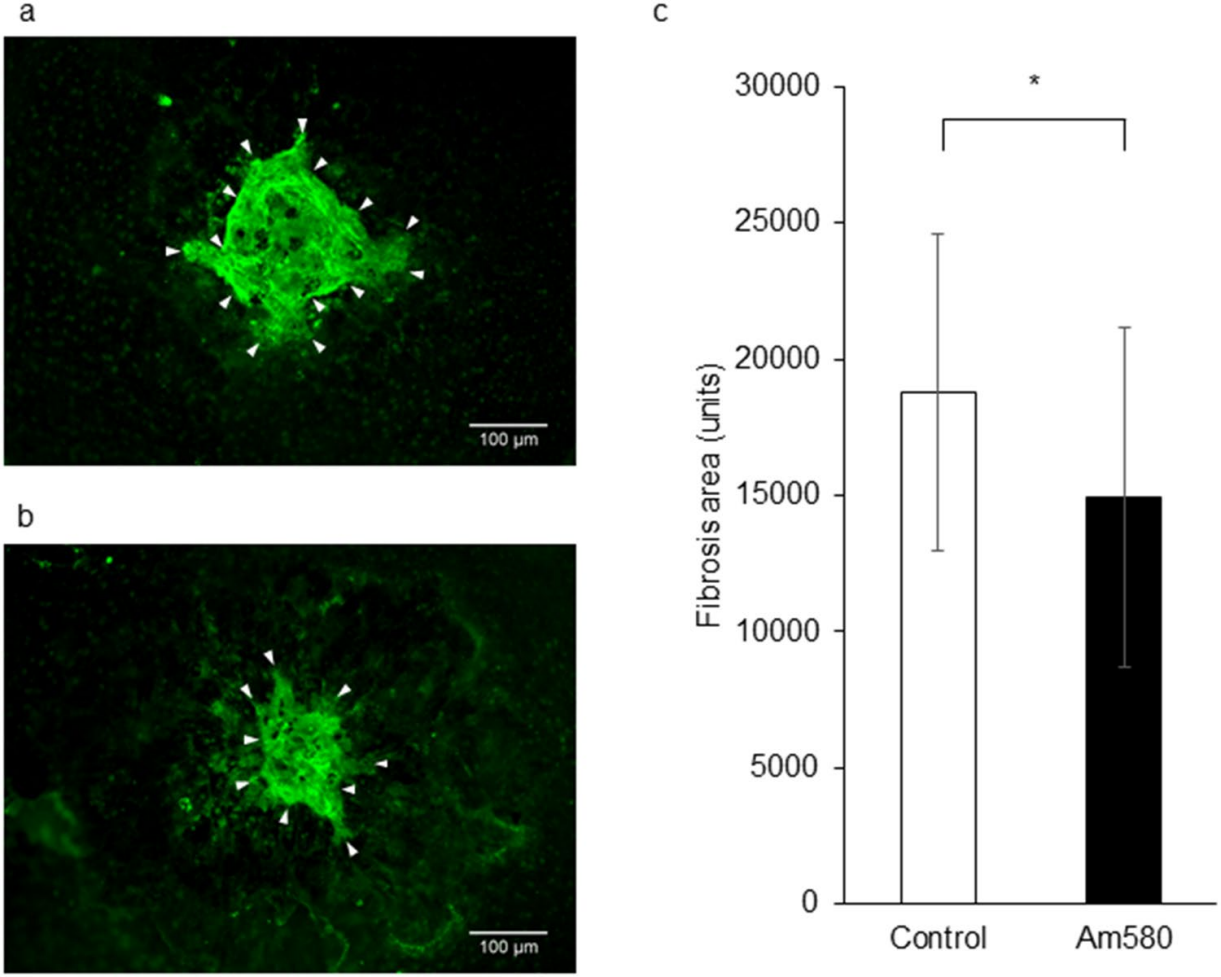
Inhibitory effect of an RAR-α agonist in a mouse model of subretinal fibrosis. Intravitreal injection (1 µl) of either PBS vehicle (**a**) or Am580 (50 µM) (**b**) was performed both immediately and 3 days after laser photocoagulation. Choroidal flat-mount preparations from the mice at 3 weeks after photocoagulation were subjected to immunofluorescence staining with antibodies to collagen type I (green). Arrowheads indicate photocoagulation-induced subretinal fibrosis. Scale bars, 100 µm. The area of fibrosis was measured with the use of the Measure command in ImageJ software and determined for the treated mice as mean ± s.d. values (*n* = 40 laser spots per group) (**c**). *P < 0.05 (Mann-Whitney test).

Finally, we evaluated the effect of the RAR-α agonist on the development of subretinal fibrosis in vivo. Immunohistofluorescence analysis with antibodies to collagen type I showed that intravitreous injection of Am580 suppressed the development of subretinal fibrosis induced by photocoagulation in a mouse model (Fig. 8).

## Discussion

We have here shown the effects of the RAR-α agonist Am580 in both in vitro and in vivo models of fibrotic tissue formation associated with AMD. The RAR-α agonist suppressed the TGF-β2-induced contraction of a collagen gel mediated by mouse RPE cells in a time- and concentration-dependent manner. It also inhibited the expression of the EMT markers α-SMA, fibronectin, and collagen type I at the mRNA or protein levels as well as the production of pro-MMP2 and TIMP-1 induced by TGF-β2 in these cells. In addition, Am580 attenuated the TGF-β2-induced expression of paxillin, release of IL-6, phosphorylation of SMAD2, and translocation of MRTF-A from the cytoplasm to the nucleus in RPE cells. Finally, the RAR-α agonist inhibited the development of subretinal fibrosis as reflected by the accumulation of collagen type I in mice. Our results thus show that Am580 inhibits TGF-β2-induced changes in RPE cells in vitro as well as subretinal fibrosis in vivo, and they therefore suggest that such an agent might have a therapeutic effect in nAMD.

Myofibroblasts contribute to the development and progression of fibrosis in individuals with nAMD^30^. Metabolites of vitamin A regulate various biological processes including development, cell proliferation and differentiation, and visual function^31,32^. One such metabolite, ATRA, also attenuates EMT processes in many cell types through activation of RAR-α, RAR-β, or RAR-γ receptors^15,23,24^. The binding of specific ligands to RAR-α, RAR-β, or RAR-γ has also been implicated in regulation of ECM accumulation and fibrosis^33^. Examination of the effects of the RAR-α agonist Am580, the RAR-β agonist BmS453, and the RAR-γ agonist R667 at the same concentrations on TGF-β1-induced collagen gel contraction mediated by Tenon fibroblasts revealed inhibitory actions of 36%, 12%, and 73%, respectively^24^. We previously showed that the RAR-γ agonist R667 suppressed EMT in RPE cells in vitro as well as subretinal fibrosis in mice^25^. The expression of RARs was not affected by EMT in RPE cells^25^. An RAR-α agonist was found to suppress cardiovascular tissue remodeling including the development of interstitial fibrosis^34^. In the present study, the RAR-α agonist Am580 inhibited both collagen gel contraction mediated by RPE cells in vitro and subretinal fibrosis in mice. These various observations suggest that both RAR-α and RAR-γ signaling attenuate EMT in and fibrosis mediated by RPE cells. Further studies are warranted to clarify the relation and potential interaction between RAR-α and RAR-γ signaling in subretinal fibrosis.

TGF-β activates various signaling pathways, including those mediated by SMAD proteins and MAPKs, and it thereby regulates various cellular processes such as EMT, ECM remodeling, and cell migration^15^ as well as contributes to the formation of subretinal fibrosis in nAMD and proliferative vitreoretinopathy^35,36^. ATRA has been shown to have antifibrotic effects that are mediated by inhibition of TGF-β signaling^37,38^. TGF-β and retinoid signaling pathways are intricately linked^39^. We previously showed that the RAR-γ agonist R667 suppressed TGF-β2-induced EMT in RPE cells in association with inhibition of SMAD and AKT signaling^25^. In the present study, the RAR-α agonist Am580 suppressed TGF-β2-induced phosphorylation of SMAD2. Further studies are necessary to clarify the relative contributions of RAR-α and RAR-γ to the regulation of SMAD and other signaling pathways activated by TGF-β during EMT of RPE cells and the development of subretinal fibrosis.

MRTF-A, a member of the myocardin family of transcriptional regulators, forms a complex with monomeric G-actin and is localized in the cytoplasm of resting cells. Activation of this factor results in its translocation to the nucleus, where it up-regulates the expression of various genes related to EMT or cell proliferation^26,40^. MRTF-A has also been shown to mediate the ATRA-induced neural differentiation of bone marrow-derived mesenchymal stem cells^41^. We previously showed that the MRTF-A signaling inhibitor CCG-1423 attenuated EMT in RPE cells^26^ and that an RAR-γ agonist suppressed both EMT in these cells and subretinal fibrosis in mice^25^. We have now shown that the RAR-α agonist Am580 suppressed the expression of α-SMA, fibronectin, and collagen type I as well as the phosphorylation of SMAD2 and the nuclear translocation of MRTF-A induced by TGF-β2 in RPE cells. Moreover, the RAR-α agonist inhibited the development of subretinal fibrosis in a mouse model. These results thus suggest that RAR-α signaling plays an important inhibitory role in the TGF-β-induced EMT of RPE cells. The relation between RAR-α signaling and RAR-γ signaling in EMT of RPE cells and in subretinal fibrosis associated with nAMD warrants further investigation.

RPE cells produce cytokines during ocular inflammation^29^. RA exerts anti-inflammatory effects by inhibiting the release of proinflammatory cytokines such as IL-6 and tumor necrosis factor-α^42^. The development of subretinal fibrosis in nAMD is also associated with the production of inflammatory cytokines^43^. We have now shown that the RAR-α agonist Am580 suppressed the release of IL-6 from RPE cells induced by TGF-β2, suggesting that this effect might attenuate the development of inflammation and consequent subretinal fibrosis.

EMT is associated with changes to the cytoskeleton and in cell-ECM and cell-cell interactions^44,45^. Cells undergoing EMT up-regulate the expression of various ECM proteins including fibronectin and collagen type I as well as acquire motility and invasive ability in part by expressing MMPs that degrade ECM proteins^46^. The major components of ECM in subretinal fibrosis are collagen types I and IV and fibronectin, with smaller amounts of collagen types III, V, and VI also having been detected^11,12^. TGF-β2 up-regulated the expression of collagen type I and fibronectin in human RPE cells^47^. In the present study, we also showed the increase in collagen type I and fibronectin expression, which inhibited by RAR-α agonist Am580 significantly. These results suggest that RAR-α signaling might contribute to ECM dynamics in subretinal fibrosis. The activation of pro-MMP2 contributes to ECM remodeling by resulting in the degradation of gelatin, collagen, and fibronectin^48^. TIMPs are endogenous inhibitors of MMPs and thus promote the deposition of ECM. TIMP-1 inhibits the activities of various MMPs including MMP2^49^. It also induces EMT in a manner independent of such inhibitory activity^16^. This latter action is mediated by signaling molecules including FAK, PI3K, AKT, and MAPKs^16^. MMP-2 activation and TIMP-1 expression are also associated with liver fibrosis^50^. We previously showed that MMP2, MMP3, and MMP8 are activated during TGF-β2-induced EMT of RPE cells, and that this activation was inhibited by the RAR-γ agonist R667^25^. We have now shown that pro-MMP2 and TIMP-1 were up-regulated by TGF-β2 in RPE cells in a manner sensitive to inhibition by Am580. These results suggest that changes in the relative balance between MMPs and TIMPs may be an important determinant of ECM dynamics in subretinal fibrosis. In this study, the expression level of pro-MMP2 or collagen type I in mouse primary RPE cells was significantly changed by TGF-β or Am580, but is less considerable. Changes in gene expression is often affected by alteration in the environment, species or condition. Further research is needed to confirm that the changes of their expression levels have an effect on biological event.

Focal adhesions play an important role in cell-ECM and cell-cell interactions, and paxillin is a major focal adhesion-related protein that regulates cell migration and remodeling of the actin cytoskeleton^51,52^. We previously showed that the ratio of phosphorylated to total paxillin in RPE cells was increased by TGF-β2, and that this effect was inhibited by the RAR-γ agonist R667^25^. In the present study, we found that the RAR-α agonist Am580 suppressed the expression of fibronectin, collagen type I, pro-MMP2, TIMP-1, and paxillin in RPE cells induced by TGF-β2, suggesting that this agent might inhibit EMT in these cells in part by modulating outside-in signaling due to cell-ECM interactions.

In conclusion, we found that an RAR-α agonist, Am580, attenuated RPE cell contraction induced by TGF-β2 as well as inhibited the associated up-regulation of EMT markers including α-SMA, fibronectin, and collagen type I and the production of pro-MMP2. Moreover, Am580 suppressed subretinal fibrosis in a mouse model of this condition in vivo. Our findings thus suggest that further investigation of RAR-α agonists as potential agents for the treatment of fibrosis related to nAMD or other proliferative retinal diseases is warranted.

## Methods

### Materials

Dulbecco’s modified Eagle’s medium (DMEM) containing high glucose (D6429) as well as minimum essential medium (MEM), fetal bovine serum (FBS), DMEM-nutrient mixture F-12, and trypsin-EDTA were obtained from Invitrogen-Gibco (Rockville, MD, USA). Cell culture flasks (60- or 100-mm diameter) and 24-well culture plates were from Corning (Corning, NY, USA). A protease inhibitor cocktail as well as mouse monoclonal antibodies to α-SMA and to β-tubulin were obtained from Sigma-Aldrich (St. Louis, MO, USA). Native porcine type I collagen (acid-solubilized), 5 × DMEM, and reconstitution buffer were obtained from Nitta Gelatin (Osaka, Japan). Bovine serum albumin (BSA) was from Nacalai Tesque (Kyoto, Japan). Goat polyclonal antibodies to TIMP-1, recombinant human TGF-β2, and an ELISA kit for mouse IL-6 were from R&D Systems (Minneapolis, MN, USA). The RAR-α agonist Am580 was from ENZO Life Sciences Institute (Farmingdale, NY, USA). Mouse monoclonal antibodies to paxillin were from BD Biosciences (Franklin Lakes, NJ, USA), rabbit polyclonal antibodies to collagen type I were from Rockland Immunochemicals (Limerick, PA, USA), and rabbit monoclonal antibodies to SMAD2 and to glyceraldehyde-3-phosphate dehydrogenase (GAPDH) as well as rabbit polyclonal antibodies to phospho-SMAD2 were from Cell Signaling Technology (Danvers, MA, USA). Rabbit polyclonal antibodies to MRTF-A (MKL-1) were from Abcam (Cambridge, UK), Alexa Fluor 488-conjugated secondary antibodies were from Invitrogen (Waltham, MA, USA), and horseradish peroxidase-conjugated secondary antibodies and ECL Western Blotting Detection Reagents were from GE Healthcare (Little Chalfont, UK). An RNeasy Mini Kit was from Qiagen (Venlo, the Netherlands), ReverTra Ace qPCR RT Master Mix was from Toyobo (Osaka, Japan), SYBR Green reagents were from Life Technologies (Carlsbad, CA, USA), and DAPI Fluoromount-G was from SouthernBiotech (Birmingham, AL, USA). Ketamine hydrochloride was from Daiichi Sankyo (Tokyo, Japan) and xylazine was from Bayer (Leverkusen, Germany).

### Isolation and culture of mouse RPE cells

RPE cells were isolated from female C57BL/6J mice (*n* = 20, body weight of 16.7 ± 0.7 g) (Japan SLC, Shizuoka, Japan) at 6 weeks of age as described previously^53^. The cells were maintained under a humidified atmosphere of 5% CO_2_ at 37 °C in culture dishes containing DMEM (F6429) supplemented with 1% MEM and 20% FBS^25,53^.

### Collagen gel contraction assay

Collagen gels were prepared as described previously^54,55^. In brief, 24-well culture plates were coated with 1% BSA (1 ml per well) for 1 h at 37 °C. Cultured mouse RPE cells were collected by exposure to trypsin-EDTA, washed twice with serum-free DMEM/F-12, and resuspended in the same medium. Type I collagen (3 mg/ml), 5 × DMEM, reconstitution buffer, RPE cell suspension (4.4 × 10^6^ cells/ml in serum-free DMEM/F-12), and deionized water were mixed on ice in a volume ratio of 7:2:1:1 to yield a final type I collagen concentration of 1.9 mg/ml and a final cell density of 1.1 × 10^6^/ml. The mixture (portions of 0.5 ml) was transferred to the BSA-coated wells of each 24-well plate and was incubated at 37 °C under 5% CO_2_ for 1 h to promote its solidification. The collagen gels were freed from the sides of the wells with the use of a microspatula, and serum-free MEM (0.5 ml) containing test agents was then added on top of each gel. The diameter of the gels was measured daily with a ruler^25,54,55^.

### Immunoblot analysis

Cells incubated in collagen gels or in 24-well plates were lysed at 4 °C in a solution containing 50 mM Tris-HCl (pH 7.5), 150 mM NaCl, 1 mM EDTA, 5 mM NaF, 1% Nonidet P-40, 0.5% sodium deoxycholate, 0.1% SDS, 1 mM Na_3_VO_4_, and 1% protease inhibitor cocktail. The cell lysates were subjected to SDS-polyacrylamide gel electrophoresis on a 10% or 15% gel, and the separated proteins were transferred electrophoretically to a nitrocellulose membrane. Nonspecific sites of the membrane were blocked by exposure to 5% dried skim milk in Tris-buffered saline containing 0.1% Tween-20 before incubation with primary antibodies. Immune complexes were detected with the use of horseradish peroxidase-conjugated secondary antibodies and enhanced chemiluminescence (ECL) reagents. The intensity of immunoreactive bands was measured with the use of NIH ImageJ software (version 1.46r; National Institutes of Health, Bethesda, MD, USA)^56^.

### RT-qPCR analysis

Total RNA was isolated from mouse RPE cells cultured in 24-well plates with the use of an RNeasy Mini Kit and was subjected to RT with ReverTra Ace qPCR RT Master Mix. The generated cDNA was amplified by qPCR analysis with SYBR Green reagents and a StepOnePlus Real-Time PCR System (Applied Biosystems, Foster City, CA, USA). The qPCR primers (forward and reverse, respectively) were 5'-AACCCTTCAGCGTTCAGCCT-3' and 5'-TCCTCTTCACACATAGCTGGAGCA-3' for the α-SMA gene (*Acta2*, NM_007392.3), 5'-GTTCGGGAAGAGGTTGTGAC-3' and 5'-CCAATGGCGTAATGGGAAAC-3' for the fibronectin gene (*Fn1,* NM_010233.2), 5'-CTAGACATGTTCAGCTTTGTGGA-3' and 5'-GCTGACTTCAGGGATGTCTTC-3' for the collagen type I gene (*Col1a1*, NM_007742.4), and 5'-GGCATTGTGGAAGGGCTCAT-3' and 5'-ATCACGCCACAGCTTTCCAG-3' for the GAPDH gene (*Gapdh*, NM_008084.3). The abundance of α-SMA, fibronectin, and collagen type I mRNAs was normalized by the corresponding amount of GAPDH mRNA^26^.

### Gelatin zymography

Gelatin zymography was performed as described previously^56^. In brief, culture supernatants (8 μl) from collagen gel incubations were mixed with 4 μl of nonreducing SDS sample buffer (125 mM Tris-HCl [pH 6.8], 20% glycerol, 2% SDS, 0.002% bromophenol blue), and 5 μl of the resulting mixture were subjected to SDS-polyacrylamide gel electrophoresis in the dark at 4 °C on a 10% gel containing 0.1% gelatin. The gel was then washed with 2.5% Triton X-100 for 1 h at room temperature, incubated for 18 h at 37 °C in a reaction mixture containing 50 mM Tris-HCl (pH 7.5), 5 mM CaCl_2_, and 1% Triton X-100, and finally exposed to Coomassie brilliant blue^56^.

### Assay of IL-6 production

Assay of IL-6 was performed as described previously^57^. Culture medium from collagen gel incubations was centrifuged at 120 × *g* for 5 min, and the resultant supernatants were frozen at − 80 °C for subsequent assay of IL-6 with an ELISA kit^57^.

### Immunocytofluorescence analysis

Mouse RPE cells were cultured at a density of 1 × 10^5^ per 12-mm cover glass in 24-well plates for 24 h, deprived of serum for 24 h, and then incubated first with or without Am580 (10 µM) for 12 h and then in the additional absence or presence of TGF-β2 (10 ng/ml) for 24 h^26,58^. After fixation for 15 min at 4 °C with phosphate-buffered saline (PBS) containing 4% paraformaldehyde, the cells were incubated with 0.1% octylphenol ethoxylate for 5 min at room temperature, washed with PBS twice, and then exposed overnight at 4 °C to antibodies specific for MRTF-A (diluted 1:250 in PBS containing 1% BSA). The cells were then washed with PBS, incubated for 1 h at room temperature with Alexa Fluor 488-conjugated secondary antibodies (1:200 dilution in PBS containing 1% BSA), mounted in DAPI (4',6-diamidino-2-phenylindole) Fluoromount-G, and examined with a BZ-X710 fluorescence microscope (Keyence, Osaka, Japan). The intensity of MRTF-A immunofluorescence in the nucleus and cytoplasm was measured with the use of ImageJ software^26,59^.

### Induction and evaluation of subretinal fibrosis in mice

A mouse model of subretinal fibrosis was generated as previously described^26,60^. Female C57BL/6J mice (*n* = 20, body weight of 19.1 ± 0.8 g) (Japan SLC) at 8 weeks of age were anesthetized by intraperitoneal injection of ketamine (90 mg/kg) and xylazine (10 mg/kg). The retinas of mice were subjected to laser-induced photocoagulation (wavelength, 532 nm; time, 0.1 s; spot size, 75 µm; power, 200 mW). The mice were randomized into two groups of 10, and PBS (1 µl) containing Am580 (50 µM) or vehicle was injected into the vitreous cavity immediately and 3 days thereafter. Mice were maintained in cages, with at five animals per cage, at a temperature of 22–24 °C and humidity of 50–70%, and with a 12 h light/dark cycle (lights on from 8:00 a.m. to 8:00 p.m.). At 3 weeks after photocoagulation, the animals were killed by cervical dislocation, and their eyes were enucleated and fixed for 2 h on ice with 4% paraformaldehyde in PBS. Choroidal flat-mounts were prepared, washed with PBS, and immersed in 100% methanol at 4 °C for 20 min. They were then exposed first at room temperature for 1 h to PBS containing 5% dried skim milk and then at 4 °C for 24 h to antibodies specific for collagen type I (diluted 1:100 in PBS). Immune complexes were detected by additional incubation for 1 h at room temperature with Alexa Fluor 488-conjugated secondary antibodies (1:1000 dilution in PBS). After washing with PBS, the preparations were mounted in PBS containing 50% glycerol and were then observed with a BZ-X710 fluorescence microscope (Keyence). The area of subretinal fibrosis was measured with the use of ImageJ software^26,60^.

### Study approval

All animal experiments were approved by the animal ethics committee of Yamaguchi University Graduate School of Medicine and were performed in accordance with the relevant guidelines and regulations. The study is in compliance with the ARRIVE guidelines for the in-vivo studies carried out on animals.

### Statistical analysis

Quantitative data are presented as means ± s.d. and were analyzed with Dunnett’s multiple-comparison test or the Mann-Whitney test. A *P* value of < 0.05 was considered statistically significant.

## Supplementary Information

Supplementary Information.

## Data Availability

The data sets generated or analyzed during the current study are available from the corresponding author on reasonable request.
