# Supplementary material for: Inhibition of epithelial–mesenchymal transition in retinal pigment epithelial cells by a retinoic acid receptor-α agonist
Source: Sci Rep. 2021 Jun 4;11:11842. doi: 10.1038/s41598-021-90618-4 (PMC8178299; doi:10.1038/s41598-021-90618-4)
Supplement: Supplementary file 1 — Supplementary Information. [file 41598_2021_90618_MOESM1_ESM.pdf]

Supplementary Information

Original Western Blots and Gelatin Zymography Gel

Inhibition of epithelial-mesenchymal transition in retinal pigment epithelial cells by a retinoic acid receptor–α agonist

Yuka Kobayashi, Kazuhiro Tokuda, Chiemi Yamashiro, Fumiaki Higashijima, Takuya Yoshimoto, Manami Ota, Tadahiko Ogata, Atsushige Ashimori, Makoto Hatano, Masaaki Kobayashi, Sho-Hei Uchi, Makiko Wakuta, Kazuhiro Kimura*

Department of Ophthalmology, Yamaguchi University Graduate School of Medicine, 1-1-1 Minami-Kogushi, Ube City, Yamaguchi 755-8505, Japan

Figure 2a.

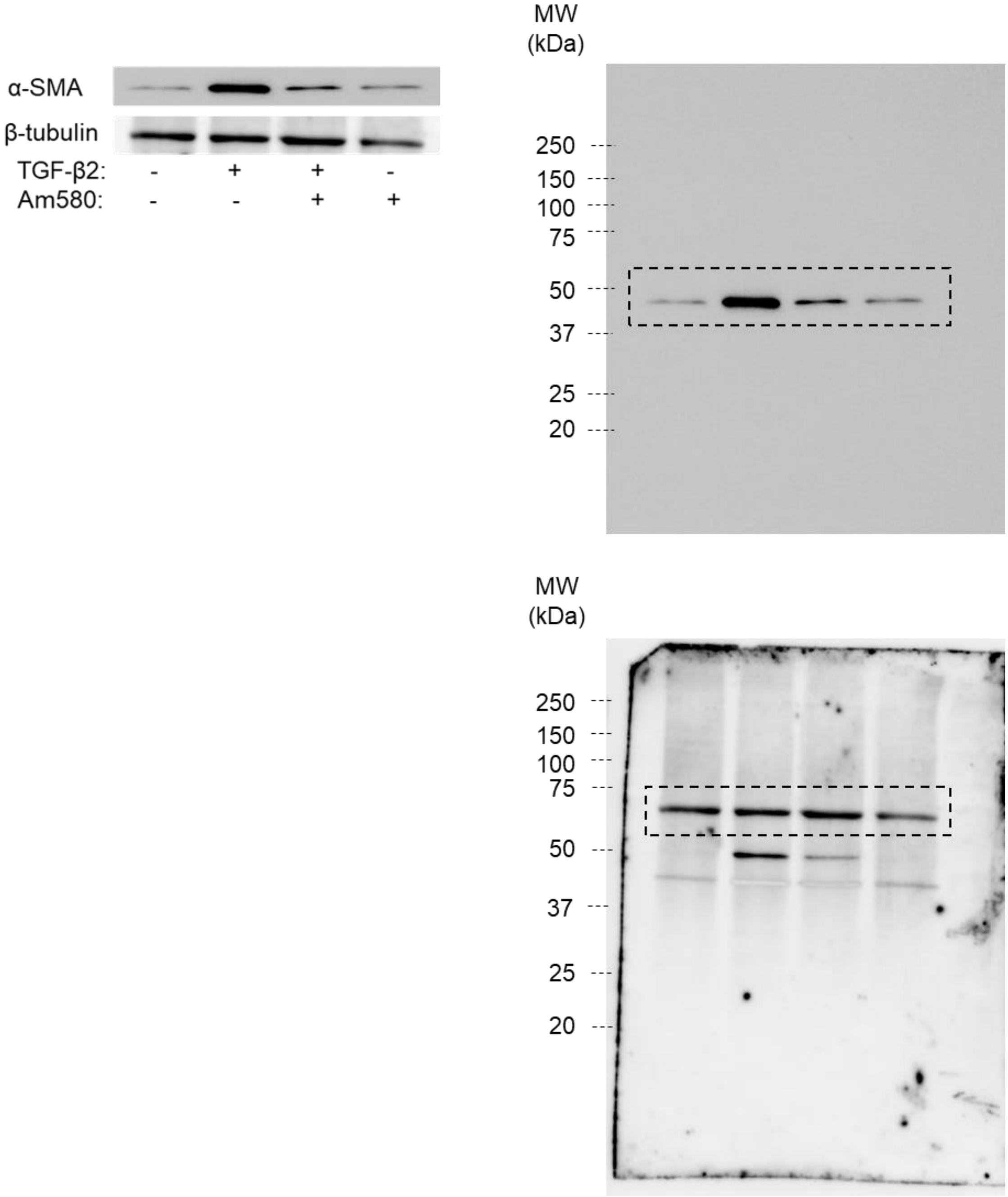

These are full length blots and the samples derive from the same experiment and the blots were processed in parallel.

Figure 3a, c.

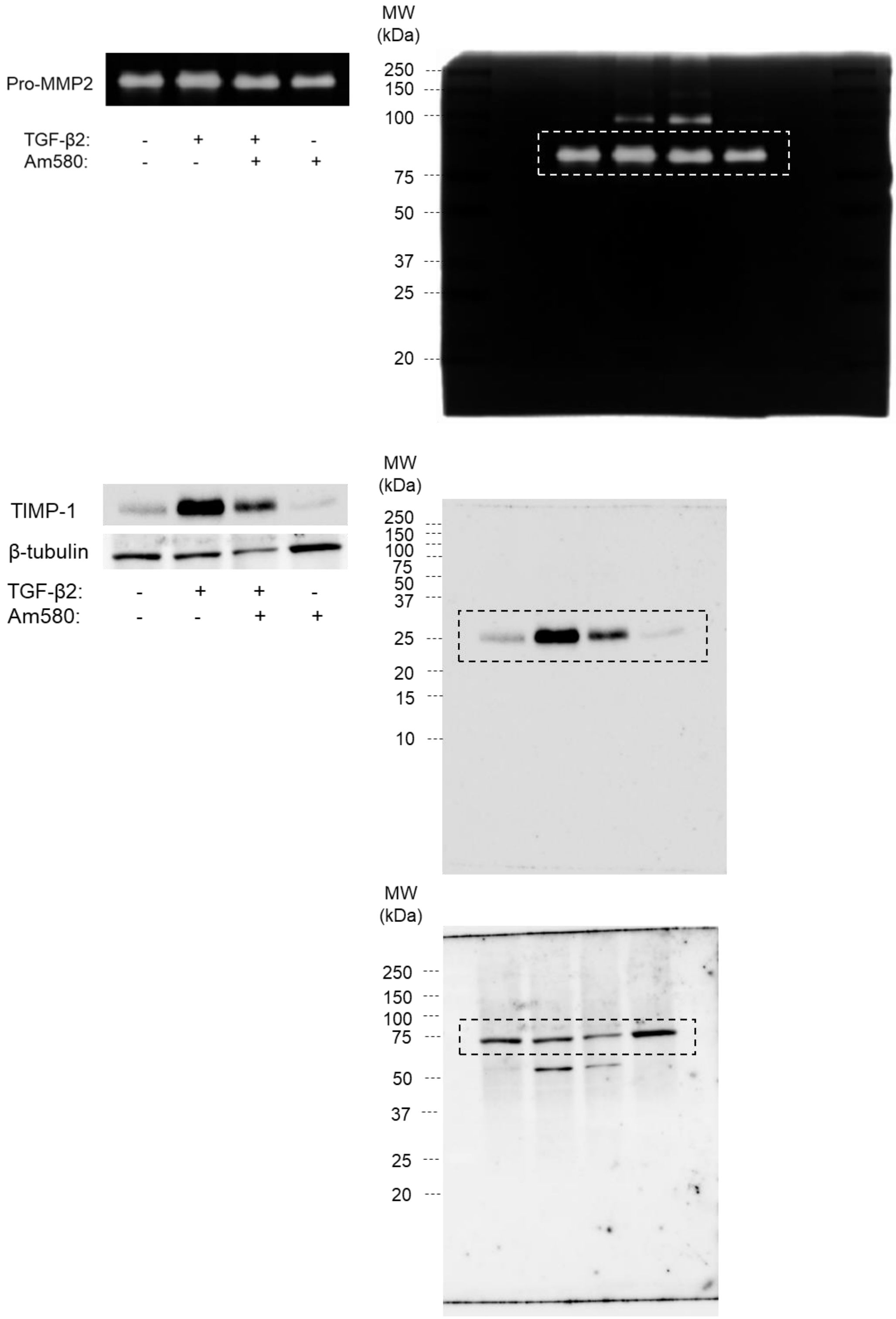

These are full length blots and the samples derive from the same experiment and the blots were processed in parallel.

Figure 4a.

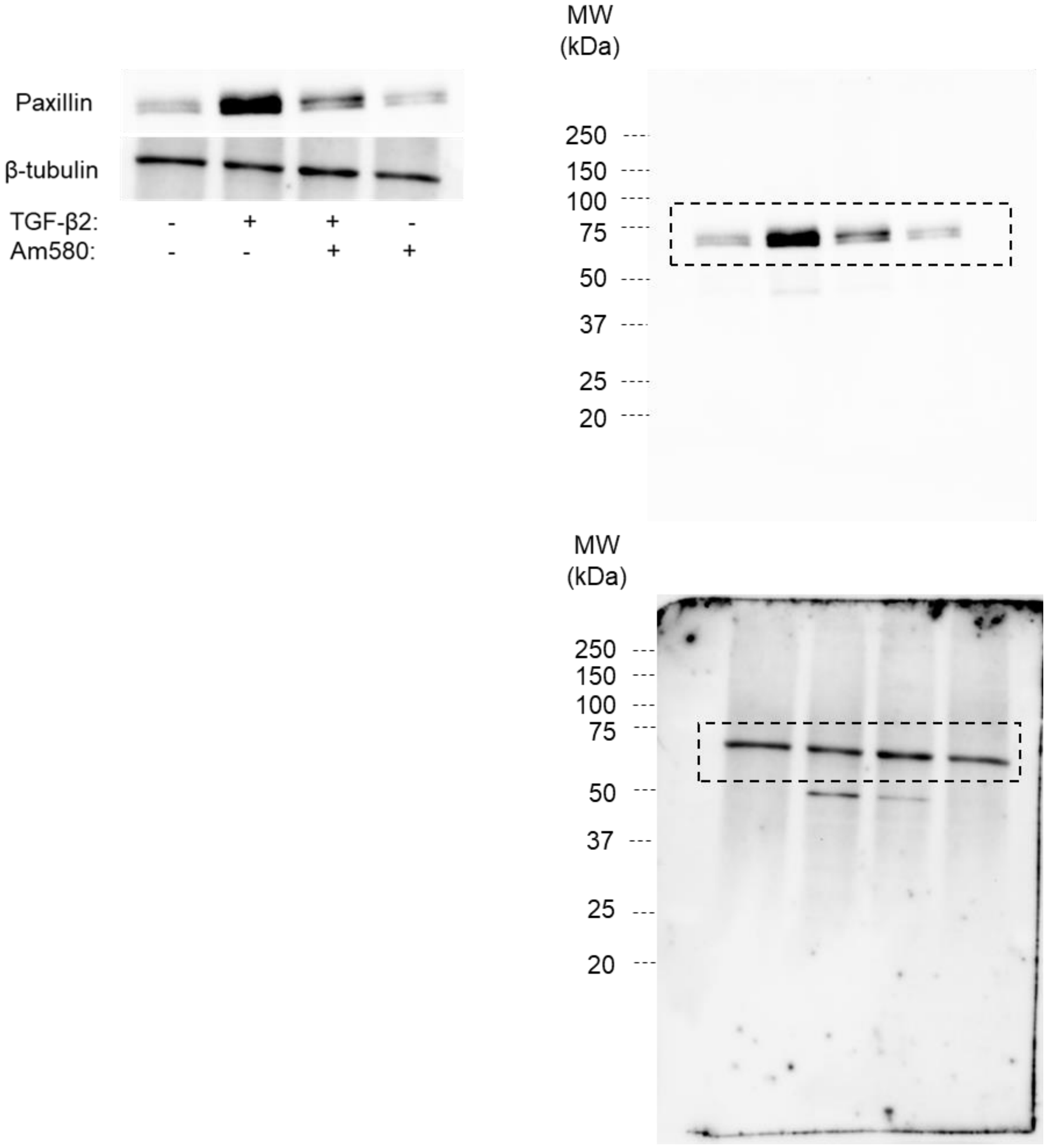

These are full length blots and the samples derive from the same experiment and the blots were processed in parallel.

Figure 6a.

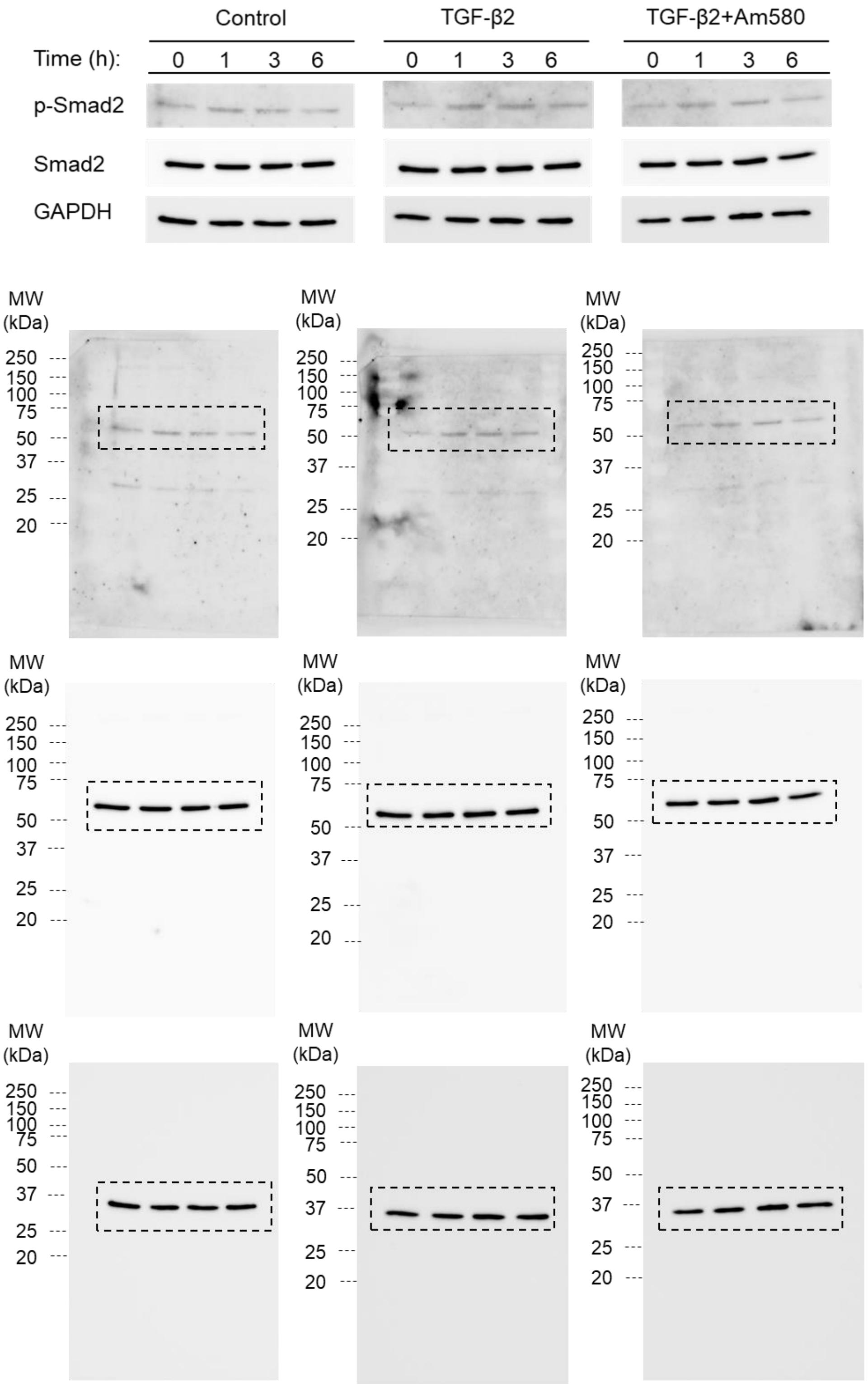

These are full length blots and the samples derive from the same experiment and the blots were processed in parallel.
